# Psychological impact of COVID‐19 pandemic and lockdown among the population involved in tourism sector in Lakeside of Pokhara: A qualitative study

**DOI:** 10.1002/hsr2.1382

**Published:** 2023-06-30

**Authors:** Rajesh Paudel, Seema Khadka, Jeetendra Rai, Rakesh Singh

**Affiliations:** ^1^ Transcultural Psychosocial Organization Nepal Kathmandu Nepal; ^2^ Helen Keller Intl Nepal Lalitpur Nepal

**Keywords:** COVID‐19, mental health, Nepal, tourism

## Abstract

**Background and Aims:**

The COVID‐19 pandemic has exerted a substantial influence on every sector of people's lives worldwide, including Nepal. The tourism industry is not exceptional. Lakeside Pokhara is one of the country's major tourist hubs and relies both on national and international visitors. The people residing in this area who depend on tourism‐related businesses to regulate their daily living faced numerous stressors and psychological impacts due to the pandemic. This study aimed to explore the COVID‐19 pandemic‐related stressors and their psychological impact among people dependent on the tourism business in the Lakeside of Pokhara, located in the Gandaki Province of Nepal.

**Method:**

Via the qualitative approach, semi‐structured in‐depth interviews were conducted to collect the data from 20 individuals related to tourism business stakeholders in Lakeside of Pokhara. Thematic analysis was performed to analyze the data.

**Results:**

The study found business‐related stressors among the people dependent on the tourism businesses, and these stressors were found to increase the experiences of psychological issues, including suicidal ideation. The pandemic has not only affected their economy but has also had an impact on their personal, familial, and social life. However, to combat the problems most of the study participants were found to be utilizing positive coping mechanisms, whereas some respondents were observed to consume more alcohol as a negative coping strategy.

**Conclusions:**

People indulging in the tourism sectors were at greater risk of vulnerability in the future pandemic. Tourism business stakeholders struggled to combat the numerous stressors and psychological impacts carried out by the COVID‐19 pandemic and lockdown. Therefore, there is a growing need for government bodies to implement favorable business‐related policies, and Mental Health and Psychosocial Support (MHPSS) related programs to these stakeholders.

## 1 BACKGROUND

A novel coronavirus named severe acute respiratory coronavirus 2 (SARS‐CoV‐2) was first identified in Wuhan City, Hubei Province in China, at the end of 2019.^1^ Since then, it has infected over 173.2 million people in the world. As of January 25, 2023, there have been 664 million confirmed cases of COVID‐19 and almost 7 million deaths.^2^ The first case of COVID‐19 was identified on 23 January 2020 in Nepal and the second case on March 24, 2020.^3^ After confirmation of the second case, the Government of Nepal announced a nationwide lockdown from March 24, 2020. Even domestic and international flights were stopped to prevent the entry and spread of COVID‐19 into the country.^4^ Similarly, Nepal was hit by a second wave of COVID‐19 in mid‐April 2021 and the daily infection rate intensely increased to almost 10,000 persons per day from 300+ persons per day within a month. As a result, the Government of Nepal decided on a national‐wide lockdown from April 21, 2021.^5^ All the transportation systems, including flights and other business‐related activities were deserted as in previous lockdowns.

The COVID‐19 pandemic caused huge disruptions in almost all industries in Nepal and tourism being one of the largest industries of Nepal was not an exception. It was estimated that Nepal has lost more than $300 million due to the lockdown enforced in the wake of Covid‐19.^6^ Similarly, COVID‐19 related restriction led to plunged Gross Domestic Product (GDP) and by a steep 46.6 percent in 2020.^7^ Tourism revenue in 2019 accounted for 7.4% of the country's GDP and supported more than 1.16 million jobs, with the expectation of providing more than 1.35 million jobs by 2020, however, only 1.09 million jobs were created in 2020 and 1.06 million in 2021.^7^

With the onset of 2020, Nepal government had planned to launch an ambitious tourism campaign “Visit Nepal Year 2020” with the theme of “Life time experience” and for this government had targeted two million international tourists by the end of 2020. To acknowledge the campaign, tourism stakeholders had planned to positively affect infrastructure development and create huge public and private investments in the tourism and hotel sectors in 2020.^8^ Unfortunately, the campaign had been postponed due to the COVID‐19 outbreak, which led to declining tourist arrival in the country. While compared to the previous numbers of tourists in 2019, the decreasing rate of tourists in 2020 was 1.96% in January, 1.00% in February, and 73.26% in March.^9^

Mental health is “a state of well‐being in which the individual realizes his or her own abilities, can cope with the normal stressors of life, can work productively and fruitfully, and is able to make a contribution to his or her community.”^10^ The effect of the current COVID‐19 pandemic on global mental health has not been registered and measured sufficiently. While COVID‐19 is likely to bring both short‐term and long‐term mental health consequences, but the existing research findings has majorly focused on immediate mental health issues; therefore, it urges more in‐depth studies on COVID‐19 and mental health.^11^ A study conducted among 1257 healthcare workers in China found that more than half of the healthcare providers had experienced the symptoms of depression, anxiety, insomnia, and distress.^12^

The COVID‐19 pandemic will substantially involve different phases with regard to mental health.^13^ The first is the acute phase when people become aware of national and local causes, and steps are taken to limit the spread of the disease. Changes like lockdowns and school closures can trigger acute stress reactions and adjustment issues and can exhibit insomnia, paranoid traits, and disruptive behavior as they attempt to cope with the strain. Suicidal feelings have also been reported as the cause of the pandemic. This is followed by the sub‐acute phase.^14^ It has been anticipated that the global impact of COVID‐19 is likely to last for several years.^15^ People need to adapt to the new changed socio cultural circumstances, and this could result in stress, pathological habituation, anxiety, and more.

Mental health conditions after the COVID‐19 pandemic has become the global concern. A survey conducted on the psychological impact of lockdown in Nepal suggests that about one‐fourth of the responders had prominent anxiety (25.4%) and 7% reported depressive symptoms.^16^ On the other hand, suicide became a major issue during the COVID‐19 outbreak. During the first wave of lockdown in Nepal, the prevalence of suicidal behavior across nation compared with all of the previous year was relatively high.^17^ Similarly, the health care professionals who worked in the health facilities of Nepal observed more self‐harm and suicidal tendency during outbreaks with a rate of 3%−5%.^17^ Similar, to other people, tourism‐related professionals too should have been affected psychologically, but has remained to be unnoticed due to lack of research among this specific population. Hence, this study aimed to explore the COVID‐19 pandemic‐related stressors and their psychological impact among people dependent on the tourism business in the Lakeside of Pokhara, located in the Gandaki Province of Nepal.

## 2 METHODS

The study was a cross‐sectional qualitative study. The approval for conducting the study was obtained from the internal research committee of the Department of Psychology and Philosophy, Tri‐Chandra Multiple Campus, Tribhuvan University (Ref. No. TC‐Psy‐2021122‐047). Concerning the COVID‐19 protocols, verbal consent was obtained from each participant before the commencement of the interview.

### 2.1 Study settings

The study was conducted in the Lakeside area (Ward no. 6) of Pokhara, Gandaki Province, located in the western part of Nepal. It has a total population of 14,735 where 7008 are males and 7727 are females with a 3869 household population.^18^ Lakeside is the hub for hotels, restaurants, travel essentials and trekking equipment shops. The business in the area heavily rely on domestic and international tourists.^19^ The district in the province had on top of the list of active cases of COVID‐19 during the period of July 2021. The most of infected and the deceased are from the Pokhara Metropolitan City where the study area belongs.^20^ The reasons for COVID cases were due to relaxing prohibitory orders and decreasing adherence to public health and social measures.^21^

### 2.2 Research participants

A total of 20 participants were interviewed purposively. By the time we interviewed 20 participants, there was no new information coming indicating the data saturation. The sample characteristics of the participants are provided in Table 1.

**Table 1:** Sample characteristics of the participants.

Participant No.	Sex	Age	Education level	Nature of business involved	Position at business
P1	Male	45	Bachelor	Hotel and Restaurant	Owner
P2	Male	36	+2	Art and Handcraft	Owner
P3	Male	39	+2	Hotel and Restaurant	Owner
P4	Female	42	Bachelor	Art and Handcraft	Owner
P5	Male	43	Master	Adventurous Tools Center	Owner (Trade Union Leader)
P6	Male	33	Bachelor	Hotel and Restaurant	Owner
P7	Male	41	+2	Tour and Travel	Owner
P8	Female	37	Bachelor	Hotel and Restaurant	Owner
P9	Female	44	+2	Hotel and Restaurant	Owner
P10	Male	28	Bachelor	Art and Handcraft	Owner
P11	Male	49	+2	Hotel and Restaurant	Owner
P12	Male	51	School Level	Tour and Travel	Driver
P13	Male	55	Bachelor	Hotel and Restaurant	Owner
P14	Female	36	+2	Hotel and Restaurant	Owner (family Business)
P15	Male	37	Bachelor	Art and handcraft	Owner
P16	Female	33	Bachelor	Tour and Travels	Manager
P17	Male	25	Bachelor	Art and Handcraft	Owner (Family Business)
P18	Male	53	Bachelor	Hotel and Restaurant	Manager
P19	Female	26	School Level	Hotel and Restaurant	Waiter
P20	Male	48	+2	Adventurous Tools Center	Owner

### 2.3 Data collection

In‐depth interviews were done using semi‐structured interview guideline. All interviews were conducted in July 2021 using face‐to‐face interview technique. To avoid the risk of COVID‐19 transmission, appropriate precaution measures were taken as suggested by WHO, and the Ministry of Health and Population (MoHP), Nepal.^22^ Each interview lasted for about 45 min on an average. The interviews were conducted in Nepali language. Informed verbal consent was taken with each participant before the interview considering the COVID‐19 protocols. Among them, seven participants agreed to have their interview audio recorded, whereas the remaining 13 interviews were noted in the notebook. Recorded interviews were transferred to a password‐protected computer, followed by transcribing of the data, and the notebooks used to note the interviews were immediately checked for completeness. The interviews were then translated into English for the analysis purpose. R. P. and J. R. checked the quality and completeness of the translations independently.

### 2.4 Data analysis

The data was analyzed manually using a thematic analysis approach.^23^ Following the translation of the fifteenth interview, data analysis commenced. Two authors, R. P. and J. R., undertook independent coding of the first five interviews. Upon reaching a common consensus, subsequent interviews were coded separately by each author. The analysis was performed by categorizing the codes into three major themes (increase in business‐related stressors, mental health vulnerability, and resilience).

### 2.5 Findings

The findings of the study are organized in three major themes, which are further subdivided into sub‐themes.

### 2.6 Theme A: Increase in business related stressors

Sub themes: 1. Financial burden and its effect on family members.

2. Managerial issues.

#### 2.6.1 Theme B: Mental health vulnerability

Sub themes: 1. Suicidal risk behavior triggered by mental distress.

2. Lack of knowledge of mental health well‐being and help‐seeking behavior.

#### 2.6.2 Theme C: Resilience

##### Theme A: Increase in business related stressors

The COVID‐19 pandemic and subsequent lockdown measures have acted as a catalyst for an increase in stress among the general population. Both job seekers and business traders were experiencing various stressors due to the pandemic, with the severity and nature of these stressors proving to be unbearable. Common stressors among the business person included bank loans, government taxes and house rent, but newcomers in the business had been affected by the stressors as they have started business with the zero bank balance hoping for the profitable business in the future. Among these individuals, some participants expressed concern about not being able to meet their monthly loan payments.

“It has been less than four months since the business was established, and shortly thereafter, the government ordered a lockdown resulting in the store's complete closure. Additionally. the cost of rent is expensive. We have been unable to meet these expenses. The business had started with a zero bank balance, relying solely on bank loans for investment. Consequently, the bank installment remains unchanged despite the current circumstance.” (Participants 1, 45 years, Male, Lakeside Pokhara)

One participant expressed their distress regarding bank loans and their regular settlement system. He expressed his feeling in a way that suggested he had no other option, which led to an overwhelming sense of distress.

“Regardless of whether our business is operational or not, we are obliged to make payments to both the bank and the taxes to the government. However, we are facing challenges to comprehend the reasoning behind this requirement.” (Participants 2, 36 years, Male, Lakeside Pokhara)

Sub theme 1. Financial burden and its effect on family members.

The impact of financial burden in business is extend beyond individual stress and can have indirect effects on family relationship and dynamics. Some participants in the study reported that their distress was more likely to be displaced to the family, particularly when spending time together, leading to conflict between couples. Additionally, families are struggling to meet the financial demands of their children, including regular expenses and school fees.

“Before lockdown, we used to work from office but in the past few month the COVID‐19 forced us to stay at house with family. The family relationship was quite good previously, but spending all our time together has caused some misunderstanding to arise. Moreover, we must still consider school fees for our and adhere to family regulations as we did before, but the current situation is highly unfavorable for us.” (Participants 3, 39 years, Male, Lakeside Pokhara)

The ongoing crisis in the market has resulted in cessation in regular income for many individuals and has caused a notable increase in daily expenses. To make matters worse, certain bank balances that were saved for the future are now being depleted. Meanwhile, some participants expressed concern over their children's lack of awareness regarding their family's financial situation. A female participant shared her experience as:

“How beneficial would it be for us to consistently utilize our saved money throughout the year? We must also consider the future ahead. We, as a parents can understand the problem, but explaining it to our children is challenging. Children want the things to remain regulated as they were in the past, however, the circumstances have changed.” (Participants 4, 42 years, Female, Lakeside Pokhara)

In addition to the financial constraints, many participants in this research identified the risk of damage to goods and items in their stores as an emerging issue for traders. One participant who operated an adventure‐related business shared his perspective, stating:

“Tourism is a highly expensive industry, often associated with adventurous pursuits such as rafting, trekking, climbing, and jumping. The equipment required for these sorts of activities are quite expensive and can deteriorate rapidly if it left unused. Most of the items have already been ruined, while a few others are currently deteriorating. The machinery part of this equipment is not working properly because of excessive rust in knot bolt parts”. (Participants 7, 41 years, Male, Lakeside Pokhara)

Sub theme 2. Managerial issues

The study participants also reported that the employers are facing significant challenges. These include difficulty in paying workers on time, the loss of skilled workers, and the uncertainty regarding the future of business. One participant, who worked for a large and reputed hotel, described managing employees in a current situation as the most stressful aspect of their job.

“Once they join as an employee of our organization, we perceive them as member of our own family. Previously, we had a workforce of 150 individuals until COVID‐19 affected us, drastically reducing it to just fifteen. The workers who have been working with us are not even paid on time. The rest of the workers reach out me daily, but regrettably, I am currently unable to respond their inquiries.” (Participants 18, 53 years, Male, Lakeside Pokhara)

As a result of the trade closure, many merchants have been forced to reduce their staff, and those who remain are often receiving unsatisfactory compensation. Traders are also worried about the future of their business once normal situations resume, particularly since they have already had to let go of skilled workers due to financial strain. One participant, who operates multiple business in the lakeside of Pokhara, described her situation as follows:

“In many ways, the pandemic hit the world hard, but businessmen like us are facing more challenges. I have three hotels and restaurants along with a travel agency company. To put it another way, they are all completely relevant to tourism. We have already laid off nearly 70% of our workforce, and the process will continue in the current situation. The loss of skillful employees is a problem; at the same time, it would be very difficult to recruit them just after the new normal situation. Both losses in income and loss of employees are equally devastating in the tourism business.” (Participants 6, 33 years, Male, Lakeside Pokhara)

COVID‐19 pandemic has resulted in stress for many business stakeholders, although, the specific causes of stress can vary depending on the nature of their business and their role within it. Merchants, for example, may experience stress related to issues such as salary, bank loans, house rents, government taxes, and damaged to goods inside the store. Workers, on the other hand, may be stressed by job risks, uncertainties, and family responsibilities.

#### 2.6.3 Theme B: Mental health vulnerability

The participants reported suffering from various psychological distress due to business‐related stressors such as disturbed sleep patterns, overthinking, changes in appetite, and other family and social problems.

Sub theme 1. Suicidal risk behavior triggered by mental distress

Study participants reported that they were struggling with psychological difficulties and experiencing behavioral issues in their daily lives during pandemic. Common problem cited by participants included sleeping problem, problem in social relationship, and overthinking. One participant shared his personal experience with these issues:

“Shortly after lockdowns, I felt quite difficult for some days. It was hard for me to keep up with my daily routine and other social relationships in a way I cannot even imagine now. I could not sleep until late at night, I came back here and there, and there were many things on my mind to think about. There was a time I asked myself questions about whether I needed any professional psychological help or not. However, later on, I was able to manage these goosebumps situations, somehow. Yeah, I didn't think that I managed them completely because I realized I felt the same situation in recent days if I was exposed to a long‐term stressful situation the whole day”. (Participants 3, 39 years, Male, Lakeside Pokhara)

Some participants appeared reluctant to respond to the question related to psychological distress. Nevertheless, they noticed their business friends experiencing both long‐term and short‐term emotional problems.

“The losses in my business are countless compared to the big parties of Lakeside‐Pokhara. It all depends on the bank loan. In particular, the lockdown has a tremendous effect on that new businessperson. I heard many of them are already in consultation with doctors because of emotional problems like depression and anxiety”. (Participants 7, 41 years, Male, Lakeside Pokhara)

Additionally, excessive alcohol use was reported as a factor that may exacerbate mental health issues, with some participants reporting increased alcohol consumption during the lockdown period, including individuals who had just recently started drinking.

“Mentally, our business friends are in a state of disintegration and various events are in danger of happening. I have seen that even unaccustomed friends who did not drink are used to consuming more alcohol lately. It can also be said they are using it to overcome stress”. (Participant 5, 43 years, Male, Lakeside Pokhara)

As a result of emotional disturbance and increased alcohol consumption, some individual experienced suicidal thought, leading to increase in the number of people seeking professional psychological support. A female participant described the real‐life incident of a well‐known businessperson attempting suicide near the lakeside of Pokhara.

“Trade deficits have played an important role in increasing mental health problems. Some friends have taken medication for depression. An incident happened near the lakeside just before yesterday when a reputed businessperson was rescued while trying to commit suicide. Business seems to be the cause of the problems”. (Participant 8, 37, Female, Lakeside Pokhara)

Moreover, another participant, who is involved in the art and handcrafts industry, shared her personal experience during the lockdown.

“About two years ago, I was diagnosed with depression, coinciding with my divorce from my wife. Gradually, I was making progress towards recovery, which was aided by my business and my love for painting. However, when the government announced a nationwide lockdown, the market for my business came to a halt, and my motivation towards it diminished. Furthermore, I neglected to allocate time for art at home. This period proved to be extremely challenging for me. I found myself wishing to avoid any responsibilities, spending most of my time confined to my bed. I felt a sense of worthlessness towards my profession, and my interest in practicing painting dwindled. It became apparent that I had experienced a relapse of my depression and started having thoughts of self‐harm”. (Participant 15, 37, Male, Lakeside Pokhara)

Sub theme 2. Lack of knowledge of mental health well‐being and help‐seeking behavior.

Participants who have suffered from the emotional problem visited the doctor for the consultation, however, adherence to the treatment procedure and the stigma surrounding mental health medication. They often discontinued medication and follow‐up session as suggested by their doctor. Furthermore, many participants were unaware of when and how to seek mental health treatment, leading to delays in regular follow‐up meetings. A female participant shared her experience of mental health condition and treatment as:

“It was the initial phase of the first lockdown; I began to stress about rethinking the current business. The stress later led me to depression and compelled me to consult with the doctor. The doctor diagnosed the depression, prescribed medicine for a month, and scheduled the next follow‐up meeting after that. I continued medicine for the first two months and was quiet when I was relived. I did not even visit for the next follow‐up meeting. Now I am in a better mood than in that period. However, I feel difficult emotions sometimes and I am managing them anyway.” (Participant 9, 44 years, Female, Lakeside Pokhara)

Many participants expressed their belief that they had no emotional and financial support from anyone, which prevented them from seeking help or sharing their problems with others.

“As it is with myself, it would be better not to share your bad experience with anyone. They will only laugh at you regardless of support. You should bear how much you have for yourself.” (Participant 10, 28 years, Male, Lakeside Pokhara)

#### 2.6.4 Theme C: Resilience

Many participants found themselves in stress from the start of lockdown. However, they managed to adopt positive thinking and expect something good in the near future. To handle the unprecedented situation, participants employed a variety of coping mechanisms, including problem‐solving, adopting an optimistic attitude, engaging in spiritual activities, seeking social and emotional support and validation, as well as turning to alcohol consumption and gambling. Similarly, some of the participants believed that the scenario is not for a single person but for most populations who have faced the pandemic and lockdown. A female participant shared her experience of positive coping by engaging the cultural rituals and spiritual activities.

“It would be easy to accept if we think it was not only our problem. This is the time where all the people are struggling and one day all the problems will vanish. I have taken the help of various spiritual and psychoanalytic tutorials through my mobile phone and they help me to keep calm and patience during the stressful time”. (Participants 4, 42, Female, Lakeside Pokhara)

Despite this, it has been observed that not all individuals are handling the stressful situation in a positive manner. Some individuals were found to resort to negative coping mechanisms, such as alcohol consumption and gambling,

A businessperson shared her daily routine during lockdown as;

“I was a social drinker, actually, but from past lockdowns, the frequency is increasing. There is nothing keeping me busy like my other friends. Therefore, our friends gathered to play cards and enjoy drinking during business time. In fact, the frequency of alcohol consumption was increasing.” (Participant 12, 51 years, Male, Lakeside Pokhara)

The disruption in trade led them to limitation on their budget to regulate their normal lives as previously. However, people tend to adopt the problem‐solving approach to mitigate the impact of such difficulties. Additionally, some participants in the study reported managing these crises by reducing their daily expenses, while other, such as business people, focused on the alternative source of income that could at least generate some revenue, such as exporting handicraft‐related materials to a foreign country.

A young entrepreneur shared about the processing to the optional business as:

“Recently, I have been collaborating with a foreign businessman online. My friend who stayed in the UAE (United Arab Emirates) gave me an idea for an online business. The business is not satisfactory however, I am engaging in it for the future so that I can expand more for the same.” (Participants 17, 25 years, Male, Lakeside Pokhara)

Similarly, many participants received socio‐emotional support from their family members.

“Emotional support from family was also a major factor that helps in coping reported by”. (Participant 13, 55, Male, Lakeside Pokhara)

Participants reported that they have a trade union within the city and collaboratively, they shouted for help from the policy level for the revision of policy regarding bank loan and taxes.

A businessperson who lead the trade union shared their team effort working for the policy level to get relief from the financial burden as:

“We have sent a memorandum to the government bodies through our organization to solve our problem so that our problems can be heard regarding bank loans and tax policies.” (Participant 5, 43 years, Male, Lakeside Pokhara)

Participants in this study were observed to exhibit resilience, as they constantly attempted to solve their problems and did not give up hope. Ram (pseudonym) a 29‐years old active and friendly youth, shared that he coped with his emotional stress by confining in his close friends and family, allowing him to share the suppressed negative emotion and feel refreshed.

Finally, yet importantly, the traders were found to be coping mentally and practically until the day they were interviewed for this research. However, it is important to note that if they are forced to continue dealing with stressors fora long time, their resilience may be compromised.

## 3 DISCUSSION

Three major themes emerged from the study, which includes business‐related stressors, mental health vulnerability, and resilience. The study found that major stressors for stakeholders in the tourism industry include bank loans, government taxes, and rent for the grocery. There is limited existing literature on the stressors discussed above, but one article emphasizes that many people perceived the COVID‐19 pandemic as a greater threat to the economy than to their health.^24^

Lower income is found to be associated with incidences of COVID‐19 infections, but such economic factors are also altering many of the collateral effects of the pandemic.^25^ Another study on Brazil's franchising stakeholders also backs up these study findings that the pandemic is putting a strain on the economy. Similarly, these study findings also support by the prior studies that found lockdown and halted trade have an influence on the families of business people in family relationships and child‐rearing.^21^ In the same article, it is emphasized that the increase in economic strain during COVID‐19, besides the increase of uncertainty, have put the family under strain and besides put their relationship into a real test.

The study finding emphasizes that layoffs of skilled workers as crucial factors contributing to stress for employers. They know that such layoffs can have a detrimental impact on staff morale and commitment, and can be seen as a breach of the psychological contract between the workers and employers. Similarly, losing skilled workers would incur high costs to achieve the quality level expected by their guests.^26^

The study revealed that people are vulnerable to mental health problems due to overwhelmed stressors experienced during a pandemic. Consistent with previous studies, the pandemic has resulted in deterioration of mental health throughout the United States, with rates of adverse mental health conditions increasing by three to four times since spring.^27^ One of the major reasons for being mentally vulnerable was economic strain. This finding supports the prior study which found a positive relationship between instantaneous economic hardships during the COVID‐19 lockdown and expressing feelings of depression and health anxiety.^28^ Importantly, the strength of the link between these hardships and indicators of mental health deterioration is very dependent on how well workers do at their jobs. As they had even rescued people from the face of suicide commit, several previous studies have supported statement that the study participants reported rescuing people from committing suicide. Major stressors, such as housing instability, unemployment, and health‐related concerns carried out by this pandemic may increase perceived burdensomeness and risk for suicide.^29^, ^30^ Similarly, disaster may be associated with PTSD, anxiety, and depression among the population.^31^

From our study, it was noted that people complain about problems with sleep and eating patterns, alcohol consumption, and overthinking as well as family and social problems. Some participants expressed a sense of boredom during their work hours, while others felt a disconnection from social interactions. Additionally, several participants reported experiencing feelings of guilt and shame due to their inability to meet the financial needs of their children and families. The participants not only mentioned the typical psychological impacts of the pandemic but also shared incidents of suicidal ideation and risk, including a few recent instances of successful intervention in preventing suicides. There have been several studies to support the following finding from our study. The pandemic has resulted in increased unemployment and job insecurity,^32^ personal debt, food insecurity and mortgage^33^ and rent stress.^32^, ^34^ These all are the risk factors for poor mental health.^35^, ^36^, ^37^, ^38^, ^39^ Financial strain, indebtedness, and inability to provide materially for one's family may increase stress and household conflict, undermine personal autonomy and induce feelings of shame and guilt.^40^ Although, some of the studies are inconsistent with our study finding of the connection between financial uncertainty and, suicide and self‐harm.^41^ Several study found that in the first year of the pandemic, suicide rates appear not to have risen (and in some cases declined) in most countries for which evidence is available.^42^, ^43^, ^44^ However, there might be a risk of suicide as many countries now are realizing the socioeconomic consequences of the pandemic which belongs to the indicators of poor mental health.^35^, ^36^, ^37^, ^38^, ^39^

The findings of the study suggest that, despite some of the participants experienced mental health conditions, they did not receive the necessary mental health services. Moreover, the participants appeared to lack of awareness regarding mental health and help‐seeking behavior. Stressors, stigma, and deteriorating mental health condition might have some connection with the suicidal ideation. However, there is no such study that has established a causal relationship between COVID‐19 and suicide.

The third and final theme of our discovery was resilience. Participants reported several behavioral, cognitive, and emotional coping methods, along with family and social support as resilience. Most participants were affected by the pandemic and tended to generalize their experiences to the entire population currently facing it. As a result, they exhibited the degree of optimism regarding their business losses and the economic strain they were under. The finding backed up by the study suggests optimism is one of the strong factors that can promote resilience during difficult times despite the lack of identified factors promoting resilience, including personality as well as external factors such as social and interpersonal resources.^45^ Similarly, another systematic review on the resilience of entrepreneurs in times of crisis identified factors such as attitudes adopted towards the crisis, the characteristics of the business and the entrepreneur, the relationship with institutions, human and social capital, and strategic management.^46^

Family and social support were seen as the other important factors associated with resilience. A higher and more significant perception of social support is associated with a reduced likelihood of developing psychological distress and psychiatric conditions. Adequate social support for the general population with regard to specific at‐risk populations should be provided by offering targeted, tailored messages according to the most reliable scientific evidence.^47^ Previous literature emphasizes the role of employing distraction strategies such as laughing and having fun, reducing social isolation and communicating through social media.^48^, ^49^ In our findings, participants were found to have an increased rate of alcohol consumption as a coping mechanism to tackle business‐related stress. The media survey done by TPO Nepal during lockdown found that people are consuming alcohol as a coping mechanism, which has further added to the existing mental and psychological distress.^50^ Similarly, the findings are consistent with research conducted on US adults, which found an increase in the rate of alcohol consumption as the COVID‐19 lockdown began.^51^ Comparisons before and during the COVID‐19 pandemic were made on the number of days of any alcohol use and heavy drinking and the average number of drinks consumed over the past 30 days.^51^

To sum up our study findings, common business‐related stressors such as government taxes, bank loans, business rent, and skilled worker layoffs, and these stressors may be closely responsible for making business people mentally distressed and vulnerable. Similarly, in mentally distressed business people, insomnia, fatigue, suicidal ideation, and relapsing psychological disorders may be the most common symptoms. Meanwhile, by focusing on themselves, business stakeholders may be able to cope with stressors and mental distress. Optimistic attitudes, alternative business models, and emotional support from family may be common stress coping mechanisms.

### 3.1 Limitations

The study has some limitations. Because of the stigma associated with mental illness, there is a possibility of underreporting. However, privacy was maintained to reduce this issue. We were unable to contact those people who had closed their business completely due to pandemic effects. As the study was focused on Lakeside of Pokhara only, the findings cannot be generalized to other areas.

## 4 CONCLUSION

This study showed that tourism business stakeholders of Lakeside of Pokhara, located in Gandaki province of Nepal are at risk of psychological problems because of excessive business‐related stressors. Despite various ways to cope with stressors, the best coping mechanism for dealing with these issues were not known. There is a growing need for government bodies to implement policies regarding bank loans, government taxes, and house rent to rethink their future business. Mental Health and Psychosocial Support (MHPSS) intervention and related policies are found to be necessary to alleviate the stressors, and promote and strengthen psychosocial well‐being among the tourism business dependent stakeholders.

## AUTHOR CONTRIBUTIONS

**Rajesh Paudel**: Conceptualization; data curation; formal analysis; investigation; methodology; project administration; supervision; validation; visualization; writing—original draft; writing—review and editing. **Seema Khadka**: Formal analysis; writing—original draft; writing—review and editing. **Jeetendra Rai**: Data curation; formal analysis. **Rakesh Singh**: Conceptualization; Supervision; Writing—review and editing.

## CONFLICT OF INTEREST STATEMENT

The authors declare no conflict of interest.

## TRANSPARENCY STATEMENT

The lead author Rajesh Paudel affirms that this manuscript is an honest, accurate, and transparent account of the study being reported; that no important aspects of the study have been omitted; and that any discrepancies from the study as planned (and, if relevant, registered) have been explained.

## Data Availability

The data that support the findings of this study are available from the corresponding author upon reasonable request.
